# Healthy Lifestyle Changes Improve Cortisol Levels and Liver Steatosis in MASLD Patients: Results from a Randomized Clinical Trial

**DOI:** 10.3390/nu16234225

**Published:** 2024-12-06

**Authors:** Claudia Beatrice Bagnato, Antonella Bianco, Caterina Bonfiglio, Isabella Franco, Nicola Verrelli, Nicola Carella, Endrit Shahini, Marianna Zappimbulso, Vito Giannuzzi, Pasqua Letizia Pesole, Anna Ancona, Gianluigi Giannelli

**Affiliations:** 1 Laboratory of Movement and Wellness, National Institute of Gastroenterology IRCCS “Saverio de Bellis”, 70013 Castellana Grotte, BA, Italy; claudia.bagnato@irccsdebellis.it (C.B.B.); isabella.franco@irccsdebellis.it (I.F.); nicola.verrelli@irccsdebellis.it (N.V.); 2 Data Science, National Institute of Gastroenterology IRCCS “Saverio de Bellis”, 70013 Castellana Grotte, BA, Italy; catia.bonfiglio@irccsdebellis.it; 3 Clinical Research Unit, National Institute of Gastroenterology IRCCS “Saverio de Bellis”, 70013 Castellana Grotte, BA, Italy; nicola.carella@irccsdebellis.it; 4 Gastroenterology Unit, National Institute of Gastroenterology-IRCCS “Saverio de Bellis”, 70013 Castellana Grotte, BA, Italy; endrit.shahini@irccsdebellis.it (E.S.); marianna.zappimbulso@irccsdebellis.it (M.Z.); vito.giannuzzi@irccsdebellis.it (V.G.); 5 Core Facility Biobank, National Institute of Gastroenterology IRCCS “Saverio de Bellis”, 70013 Castellana Grotte, BA, Italy; letizia.pesole@irccsdebellis.it (P.L.P.); anna.ancona@irccsdebellis.it (A.A.); 6 Scientific Direction, National Institute of Gastroenterology IRCCS “Saverio de Bellis”, 70013 Castellana Grotte, BA, Italy; gianluigi.giannelli@irccsdebellis.it

**Keywords:** exercise, Mediterranean diet, hepatic steatosis, steatotic liver disease associated with metabolic dysfunction

## Abstract

**Background:** Steatotic liver disease associated with metabolic dysfunction (MASLD) affects up to about 30% of the general adult population and is closely related to obesity and the metabolic syndrome. Cortisol, a stress-related hormone contributing to hepatic fat accumulation and insulin resistance, also promotes progression of the disease. The study aims to investigate the impact of lifestyle modifications on cortisol levels and hepatic steatosis in patients with MASLD. **Methods:** In a 16-week three-arm randomized trial, 42 patients were randomly assigned to three groups who received dietary advice (CG), dietary advice combined with aerobic exercise (AE + DA), or dietary advice with high-intensity interval training (HIIT + DA). Before the start, after 2 months of intervention, and at the end of the project, medical evaluations, routine biochemical assessments, and psychological questionnaires were analyzed. At baseline and at the end of 4 months, hepatic steatosis was evaluated by Fibroscan^®^. **Results:** In the study population, severe hepatic steatosis (74%) and obesity (98%) were prevalent at the beginning of the study. A statistically significant (*p*-value = 0.001) reduction in circulating cortisol levels was observed over time in the two groups doing exercise, especially in HIIT + DA (*p*-value = 0.006). Hepatic steatosis, assessed by Fibroscan^®^, disappeared in 10 participants (CAP value < 248, *p*-value = 0.003). CAP values and waist circumference decreased in all groups, statistically significantly in the AE + DA group (*p*-value = 0.005; *p*-value = 0.04, respectively). **Conclusions:** The study emphasizes the benefits of combining diet and exercise in managing MASLD. HIIT + DA significantly decreased cortisol levels, while AE + DA was the most potent intervention for reducing hepatic steatosis.

## 1. Introduction

Metabolic-dysfunction-associated steatotic liver disease (MASLD), a term adopted in June 2023, was formerly known as non-alcoholic fatty liver disease (NAFLD). It is a highly prevalent condition worldwide, affecting approximately 30% of the adult population [1]. MASLD refers to hepatic steatosis associated with metabolic risk factors, particularly type 2 diabetes mellitus and overweight. It encompasses a spectrum of conditions, ranging from isolated steatosis—characterized by lipid accumulation within hepatocytes as observed in histological examination—to metabolic-dysfunction-associated steatohepatitis (MASH), which is distinguished by the presence of inflammation, and/or liver fibrosis, and it shows progression to cirrhosis and hepatocellular carcinoma in some cases [2]. MASLD is strongly associated with obesity, being one of the five diagnostic criteria. In particular, abdominal or visceral obesity contributes to an increased supply of free fatty acids to the liver, driven by enhanced hepatic lipogenesis linked to hyperglycemia and hyperinsulinemia [3]. The prevalence of MASLD increases with age, obesity (up to 90% of obese individuals), and physical inactivity [4]. For these reasons, MASLD is considered the hepatic manifestation of the metabolic syndrome [4]. Several studies have demonstrated that elevated cortisol levels are closely linked to increased hepatic steatosis [5]. Cortisol is a hormone released by the adrenal gland. It regulates the stress response through activation of the hypothalamic–pituitary–adrenal (HPA) axis [1]. It acts on the tissues and cells of the liver, muscles, adipose tissue, pancreas, etc. It intervenes in energy regulation, glucose metabolism, and behavioral processes [6]. Specifically, cortisol is known to promote hepatic lipogenesis and induce insulin resistance, both critical elements in the pathogenesis of MASLD [1]. Insulin resistance, in turn, is the central mechanism through which insulin loses its ability to regulate glucose metabolism, leading to further accumulation of liver fat [7]. In addition, cortisol, through its effect on neurotransmitters (including serotonin), can lead to changes in the emotional and psychological state [8]. Often, this state can be compromised in individuals with chronic conditions such as MASLD [9], highlighting the complexity and multifactorial nature of the condition [10]. This underscores the need to address not only the metabolic aspects of MASLD but also the psychological impact it can have on affected individuals. Lifestyle modification is currently the first-line approach for managing MASLD. It is well established that adopting a healthy lifestyle can significantly reduce the risk of developing the condition while also playing a key role in its treatment [4]. Recent studies have shown that a Mediterranean diet, particularly one with a low glycemic index, can reduce hepatic steatosis and improve the quality of life in patients with MASLD [11]. Alongside dietary interventions, various types of physical exercise have been shown to improve hepatic steatosis through different mechanisms [12]. Aerobic and resistance exercise can effectively decrease hepatic fat accumulation independently of weight loss. This benefit is driven by enhanced lipid oxidation and improved insulin sensitivity, key factors in metabolic regulation [13]. In particular, aerobic exercise (AE), which utilizes aerobic metabolism to produce energy in the form of adenosine triphosphate (ATP) and includes activities such as cycling, jogging, brisk walking, and long-distance swimming [14], has been shown to have a positive impact on several factors related to MASLD, including reducing liver fat, improving resting blood pressure, and reducing serum cholesterol levels [15]. Among various training modalities, high-intensity interval training (HIIT) has emerged as an effective strategy for rapidly increasing lipid oxidation and reducing visceral fat. HIIT has been shown to attenuate MASLD by reducing adiposity and enhancing insulin sensitivity [16]. In addition, it has been considered one of the most effective methods to improve cardiorespiratory and metabolic functions [17]. It is well established that physical exercise also affects cortisol levels, in addition to its numerous benefits in chronic diseases [18]. A regular exercise program can improve hormone balance, helping to reduce the impact of cortisol on metabolic and liver health [15]. The cortisol response to exercise is influenced by specific parameters, such as the frequency, intensity, type, and duration of the activity [19]. Studies suggest that aerobic exercise, particularly of moderate intensity, can reduce cortisol levels and improve hormonal responses to stress [18]. In contrast, intense exercise sessions are known to increase circulating cortisol, indicating that intense and prolonged exercise tends to elicit a greater release of this hormone in the short term [20]. Significant increases in cortisol levels were observed immediately after HIIT sessions and up to 60 min after exercise [21]. However, there is limited research on the long-term effects of HIIT on cortisol levels. Regular exercise appears to reduce cortisol reactivity to stress, promoting an attenuated response. This has been observed both in healthy subjects and in clinical conditions such as depression, although the changes have not always been significant compared with baseline levels [22]. It is known, in fact, that regular physical activity is also associated with enhanced psychological well-being and quality of life, including reduced symptoms of depression and anxiety. Exercise helps alleviate stress and improve mood by promoting the release of endorphins, often referred to as “happiness hormones”, which elevate mood and reduce stress perception [23].

We hypothesize that individuals with MASLD may suffer a more compromised emotional state and reduced quality of life compared to the general population. This could be due to a combination of factors, including the psychological burden of living with a chronic illness and anxiety related to disease progression. Furthermore, elevated cortisol levels may be linked to this emotional state. In this context, our study aimed to evaluate whether lifestyle modifications, particularly changes in dietary habits and the regular practice of two distinct aerobic exercise methods (AE and HIIT), could impact both circulating cortisol and steatosis hepatic levels and participants′ emotional well-being. Furthermore, by comparing exercise modalities of differing intensities, we seek to better understand how specific exercise parameters influence cortisol levels and subsequently improve MASLD outcomes. This approach is designed to identify innovative therapeutic strategies for modulating cortisol to mitigate its adverse effects on MASLD progression.

## 2. Materials and Methods

### 2.1. Participants

Participants were referred to the Movement and Wellness Laboratory at the IRCCS “S. de Bellis” in Castellana Grotte, Italy, either by their general practitioners or through an online questionnaire (Google Forms) shared on the institution′s official website and major social media platforms (Facebook and Instagram) to maximize participant reach.

Patients were enrolled consecutively, and the study was registered on www.clinicaltrials.gov (NCT06186869). The study began in October 2023 and is currently ongoing. The RCT from which this study is derived had the primary objective of comparing the effects of different types of exercise on systemic inflammatory status in individuals with obesity and hepatic steatosis.

The present results are derived from the first 42 subjects recruited between October 2023 and February 2024.

All participants provided informed consent, and the study was conducted in compliance with the Declaration of Helsinki and approved by the Ethics Committee (Protocol No. 1253/CE De Bellis, 7 June 2023).

### 2.2. Study Design

This randomized three-arm interventional study was conducted with parallel groups over 16 weeks. The inclusion criteria included (1) a body mass index (BMI) ≥ 30, expressed as weight in kilograms divided by square height in meters (kg/m^2^), or an abdominal circumference > 94 cm in men and >80 cm in women; (2) age > 18 years and <65 years; (3) and moderate-to-severe NAFLD (diagnosed at the initial visit using FibroScan^®^) [24] with a CAP score ≥ 248 [25].

The definition of MASLD restricts alcohol intake in the context of steatosis, maintaining the same limits previously established for NAFLD: less than 20–30 g of alcohol per day. [25].

The subjects should not have done more than 150 min of physical activity per week in the 6 months prior to the start of the project.

The exclusion criteria included (1) individuals with normal or underweight BMI; (2) the presence of neurological or psychiatric disorders, gastrointestinal, oncological, or cardiovascular diseases (including hypertension); (3) pregnancy or breastfeeding; (4) musculoskeletal conditions preventing physical exercise; (5) inability to quantify the degree of NAFLD; (6) lack of a medical certificate confirming fitness for non-competitive physical activity.

### 2.3. Data Collection

An initial history of potential participants was taken during enrollment, collecting information on the absence or presence of medical conditions, allergies, medication use, and risk factors (smoking, alcohol consumption, stress, and sleep). At the beginning of the project, participants were also asked about their physical activity levels using the International Physical Activity Questionnaire (IPAQ-SF) [26] and completed the EPIC questionnaire [27] to assess dietary habits.

At baseline, after 2 months, and at the end of the project (4 months), standard anthropometric measurements were obtained, including weight, height, waist circumference, hip circumference, arm, calf, and wrist measurements. After an overnight fast, blood samples were collected via venipuncture in the morning, using tubes containing ethylenediaminetetraacetic acid (K-EDTA) as an anticoagulant [13]. Stool and urine samples were also collected. Weight and height were measured with SECA instruments (model 700 and model 206; 220 cm; SE-CA, Hamburg, Germany), and standard methods were used to perform biochemical measurements.

At the beginning and end of the study only, hepatic steatosis was assessed with FibroScan^®^ [24] by the institute′s gastroenterologists, and participants completed quality-of-life questionnaires and three life satisfaction assessments, which measured various dimensions of psychological well-being. Specifically, these were the SF-36 [28], the Subjective Happiness Scale (SHS) [29], the Attachment Style Questionnaire (ASQ) [30], and the Psychological Well-Being Scales (PWBS) [31].

In addition, they received an accelerometer (ActiGraph gt9x, ActiGraph Corporation, Pensacola, FL, USA) [32], a device that records movements in three axes (x, y, z), for seven days. The accelerometer was returned to the laboratory at the end of the seven days.

**Figure 1:**
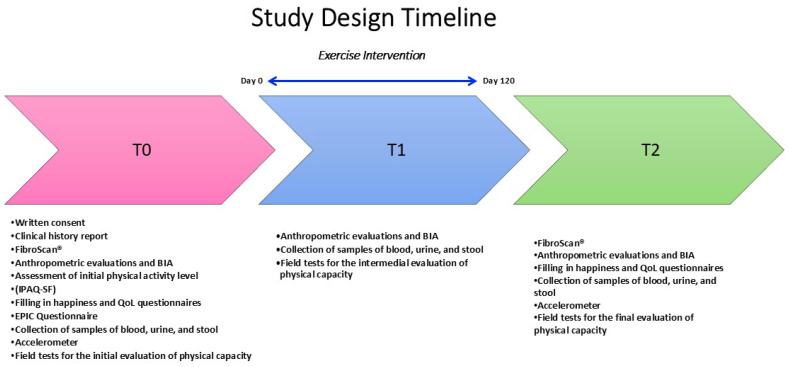
Study design timeline. BIA: Bioelectrical Impedance Analysis; IPAQ-SF: International Physical Activity Questionnaire-Short Form; QoL: Quality of Life; EPIC: European Prospective Investigation into Cancer and Nutrition; T0: Initial Evaluation; T1: Intermedial Evaluation; T2: Final Evaluation.

The study design is detailed in Figure 1.

### 2.4. Randomization and Masking

Each patient was assigned to a group based on a sequence of computer-generated random numbers. Participants were randomized to the three groups as follows: (1) the control group (CG), which followed dietary guidelines for healthy eating (CREA) [33] based on the MD with a low glycemic index; (2) the second group followed the same guidelines as the CG, in conjunction with an aerobic exercise program (AE + DA); (3) the third group adhered to the same dietary guidelines as the CG, combined with a high-intensity interval training (HIIT + DA) program.

All researchers conducting the assessments (Fibroscan^®^, questionnaires, medical history, etc.) were blinded to the group allocation. After the screening process, the statistician disclosed treatment assignments exclusively to the kinesiologists.

### 2.5. Dietary Advice

All three groups adhered to the same CREA (Food and Nutrition Research Center, Council for Agricultural Research and Economics, Rome, Italy) healthy eating guidelines, based on the low-glycemic Mediterranean diet. Participants were asked to eat a balanced and healthy diet; no guidance was given on how many calories they should consume. The prescribed guidelines were provided in brochure format, with graphic explanations of which foods to eat or not to eat (Appendix A).

Participants had the opportunity to consult with the designated dietitian if they had any questions or concerns about the dietary recommendations provided.

### 2.6. Exercise Interventions

To evaluate the initial physical condition of the patients, determine an appropriate training program, and compare baseline measurements to follow-up (FU) assessments, five field tests were conducted. Cardiorespiratory fitness was assessed using the 2 km walk test, which is suitable for adults [34]; strength and flexibility were evaluated with the hand grip test [35] and the sit and reach test [36], respectively; lower limb functionality was assessed with the Short Physical Performance Battery (SPPB) [37]; and the Timed Get Up and Go Test (TGUGT) [38] was used to evaluate dynamic balance.

The tests were performed under the same conditions, respecting time, place, equipment, and operators. All subjects wore the Polar OH1+ [39] heart rate device before, during, and after the tests.

The intervention arms that included physical exercise underwent testing at baseline, after 60 days, and after 120 days, while CG participants completed the fitness field tests at baseline and the end of the project.

The exercise interventions included two different types of programs: aerobic exercise (AE), based on a standard aerobic activity program, and HIIT, which focused on high-intensity activities. The method used to determine the intensity of the prescribed exercise was maximum heart rate (Max HR) based on Tanaka′s formula [40], assessed using heart rate monitors [41].

The structure based on the FITT parameters of the two exercise programs is shown in Table 1.

Both exercise programs were supervised by trained staff at designated times and days.

#### 2.6.1. Aerobic Exercise Program

The proposed exercise was conducted as an outdoor walk along an urban path, consisting of four sessions per week at moderate intensity (60–75% HRmax), monitored with a heart rate monitor. Each session lasted 60 min for a total of 240 min per week. All exercise sessions included warm-up, pre-exercise joint mobility, a walking period, and cool-down, including post-exercise stretching (Table 2), under the supervision of the project kinesiologists. Before starting the program, precise instructions were given about the type of shoes to wear to avoid injuries and about the functioning of the heart rate monitor.

#### 2.6.2. HIIT Program

Exercise sessions were conducted at a local gym in convention with IRCCS “S. de Bellis”, where participants completed three non-consecutive training sessions per week. Exercise intensity (≥85% maximum heart rate) was monitored continuously during each session using a Polar OH1+ [39] heart rate monitor, worn with an elastic band over the triceps and connected via Bluetooth to an iPad. Each training session lasted 50 min for a total of 150 min per week.

All training sessions began with pre-warm-up joint mobility, followed by a warm-up of about 5 min at 50–60% of FCmax, using an adapted TABATA protocol [42] (Table 3).

During the middle phase of the class, the work involved HIIT work intensity (FCmax > 85%), again using an adapted TABATA protocol [42], where high intensity was alternated with periods of active recovery. In the last mesocycle of work, we incorporated AMRAP (as many repetitions/rounds as possible) [43] to further differentiate the training stimulus.

The work included both free-body and equipment-assisted exercises. The session ended with 5–10 min of cool-down.

### 2.7. Assessment of Emotional Well-Being

Two validated questionnaires, namely the SHS, which is a 4-item self-assessment measure developed to assess overall happiness [29], and the 36-item Short Form Health Survey (SF-36), an instrument used to assess health-related quality of life, were used to assess the emotional state of the participating subjects [28]. The latter measures eight items: physical functioning (PF), physical role (RP), bodily pain (BP), general health (GH), vitality (VT), social functioning (SF), emotional role (RE), and mental health (MH) [44]. We considered only the 5 items inherent to the emotional role and happiness.

### 2.8. Statistical Analysis

Follow-up time and biological variables are presented as mean (SD) or median (IQR) for continuous variables and as frequencies (%) for categorical variables. We used the Wilcoxon rank sum test for continuous variables to compare two groups and the Kruskal–Wallis rank sum test to compare more than two groups. For categorical variables, we used the χ^2^ test to evaluate the differences.

Bar graphs were used to represent cortisol-related averages, and a box plot was used for the SHS median, stratified by follow-up time and treatment.

A generalized estimating equation (GEE) [45] was applied to estimate the longitudinal trajectories of cortisol. As outcome variables were not normally distributed, a gamma distribution (link identity) for the response was assumed, and an unstructured correlation matrix was set to the data.

GEE models are particularly useful in biomedical studies to estimate mean changes in biomarker values while controlling for covariates. They allow correlations among response data (repeated measurements on each subject) to be drawn.

Initially, confounding variables were selected from the existing literature. Then, the procedure of minimum absolute reduction and selection (LASSO) was adopted to reduce the number of candidate predictors and select those most useful for the model construction. Gender, age, ACTH, ALT, AST, and waist circumference were included as covariates. We obtained outcome predictions using post-estimation tools; this analysis is graphically displayed. The results are expressed in the natural scale of CAP measurements.

Stata statistical software v18 (StataCorp, 4905 Lakeway Drive, College Station, TX, USA) was used for statistical analyses. The official command xtgee was selected.

## 3. Results

**Figure 2:**
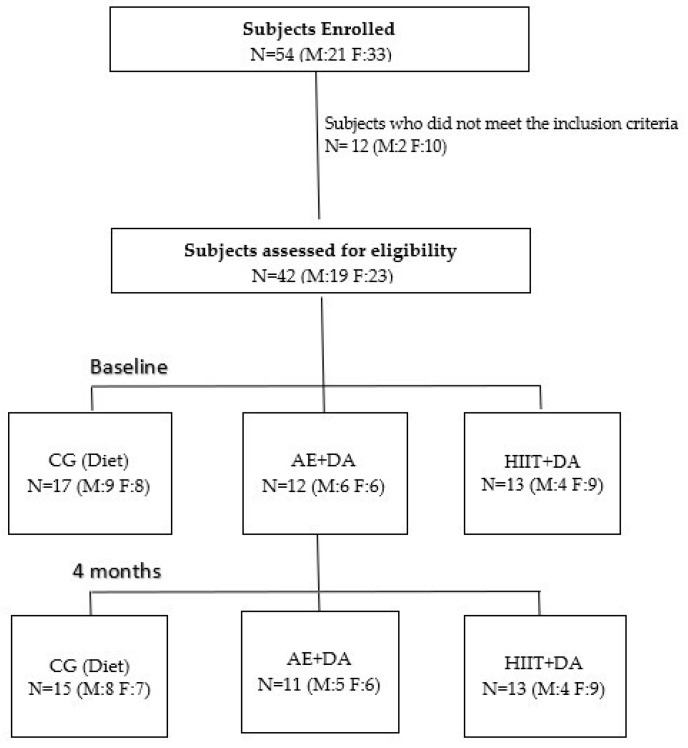
Flowchart of the participants. N: Number of subjects; M: Male; F: Female; CG: Control Group; DA: Dietary Advice; AE: Aerobic Exercise; HIIT: High-Intensity Interval Training.

Fifty-four people applied to participate in the project. Twelve subjects were excluded because they did not meet the inclusion criteria, and forty-two were included in the study. Overall, 17 subjects (40.5%) were allocated to the control group, 12 (28.6%) to the AE + DA group, and 13 (30.9%) to the HIIT + DA group. Three subjects (one female and two males) did not finish the project for personal reasons. The flowchart of the study participants is shown in Figure 2.

At baseline, 74% (*n* = 31) of subjects had severe steatosis, and 98% (*n* = 41) were classified as obese, four of whom presented severe obesity. The majority (55%) were female. The average age was 51 ± 10.52 years, with a mean of 51.52 ± 11.12 for women and 51.56 ± 9.89 for men.

Among the participants, 80.95% were non-smokers, and most (69.05%) were married or cohabiting.

The average daily consumption of alcohol grams was 8.17 (±17.14) for men and 2.60 (±3.78) for women.

No physical activity had been practiced by 58.54% of the participants, while 41.46% had done physical activity at amateur level, mainly walking.

The attendance of participants was monitored in both exercise groups (AE and HIIT) to assess adherence. Overall, 14 participants (6 in AE, 8 in HIIT) attended more than 80% of the sessions; 8 participants (4 in AE, 4 in HIIT) attended between 70% and 80%; and 1 participant (0 in AE, 1 in HIIT) attended between 60% and 69% of the sessions. Only two participants in the AE group attended less than 60% of the total sessions.

At baseline, there were no statistically significant differences between the arms, except for triglyceride values.

Table 4 shows the distribution of participants according to follow-up time, and Table 5 shows the distribution according to follow-up time and work arms.

Initial steatosis levels showed a significant improvement in 10 participants, whose CAP value decreased to less than 248 (steatosis absent).

There were also statistically significant improvements in cortisol levels over time, as the levels decreased from 16.68 (7.30) at the beginning of the study to 11.25 (3.53) at the end (*p*-value < 0.001). Furthermore, there was an improvement in waist circumference, which decreased from a mean of 106.96 (10.02) at the beginning of the study to 99.42 (13.99) at the end of the fourth month (*p*-value = 0.012).

Table 5 shows the distribution according to follow-up time and work arms.

Among the arms, subjects who received AE + DA as treatment experienced a statistically significant improvement in CAP, as the median value at baseline of 326 (296–335) decreased to 265 (246–295) by the end of the study (*p*-value 0.005).

At baseline, all participants had liver stiffness values less than 7 kPa, indicating the absence of fibrosis [25]. No significant differences were observed between groups at baseline. After 4 months, stiffness values decreased in all groups, with a more pronounced decrease in the DA + HIIT group, although this change did not reach statistical significance.

At the end of the study, serum cortisol levels had decreased in all treatment groups. Both the AE + DA and HIIT + DA groups showed a statistically significant decrease (*p*-value 0.021), with an initial mean of 17.20 (6.22) falling to a final mean of 12.16 (3.22) and a *p*-value of 0.006, and with an initial mean of 17.34 (9.09) falling to a final mean of 10.63 (3.87), respectively.

Participants in AE + DA as the treatment group had the most significant reduction in waist circumference from the baseline to the end value [from 108.12 (12.85) to 92.40 (21.54), respectively; *p*-value 0.045].

All biochemical values improved over time, regardless of the treatment arm, although no statistical significance was observed.

**Figure 3:**
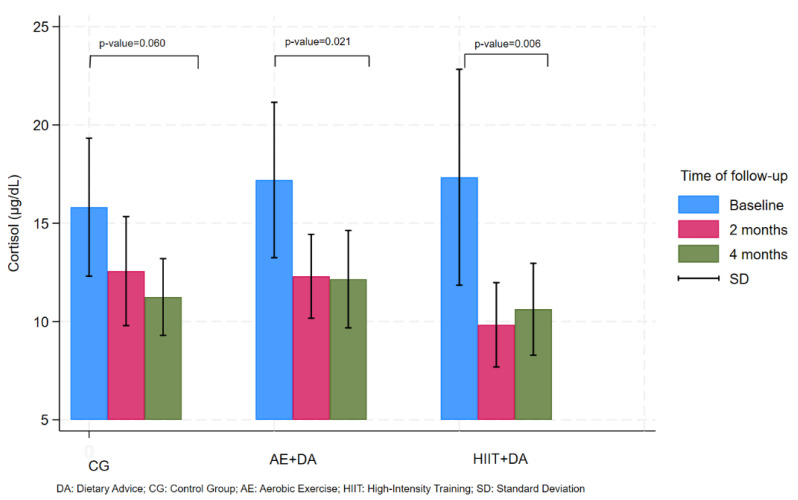
Bar graph of cortisol levels (mean and standard deviation) distributed according to time and treatment arms. The Kruskal–Wallis test was used to assess time differences among the three treatment groups.

The bar graph (Figure 3) shows the time trend of the cortisol values, observed in Table 2.

Table S1 shows the distribution of participants according to follow-up time and working arms for the eight subscales of the SF36 questionnaire [46] for the five variables related to the “emotional well-being” group and the Subjective Happiness Scale (SHS) [47]. Values are expressed as median values (IQR).

**Figure 4:**
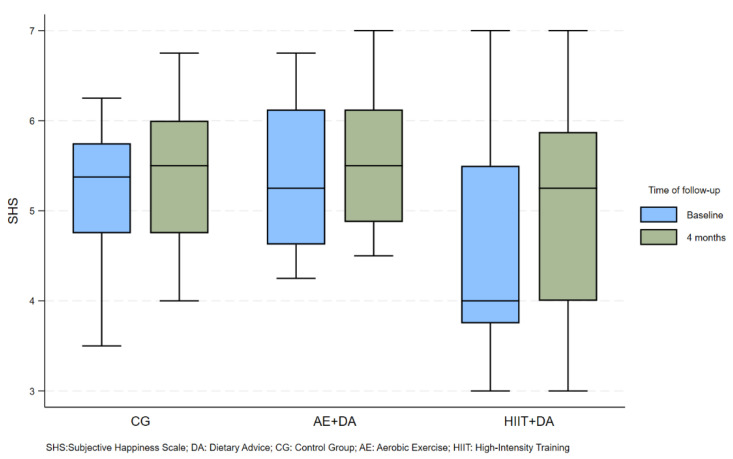
Box plot of SHS (median and interquartile range) distributed according to time and treatment arms.

The box plot (Figure 4) shows the development of the SHS value over time, as shown in Table S1. The median value of the happiness questionnaire increased slightly over time in the CG and AE + DA groups, whereas the change was more pronounced in the HIIT + DA group. However, it should be noted that the observed differences were not statistically significant.

The estimated modification effects between the working and time arms showed a statistically significant reduction in cortisol levels in all groups, as can be seen in the results in Table 6.

A statistically significant main effect of time on cortisol levels was found from the second month onwards (−5.85, 95% CI: −8.88; −2.82, −4.84, 95% CI: −7.94; −1.75).

No statistically significant main effect on cortisol levels by treatment was found.

Table 7 shows the contrast between the expected mean values for the modification effect between time and working arms at the two time points and the initial values.

Notably, the most significant reduction was observed in all groups during the second month of treatment compared to the baseline, as shown in Table 7. The HIIT + DA group exhibited the largest reduction of −11.10 (95% CI: −14.9; −7.28).

The longitudinal trajectories of cortisol levels by working arms are shown in Figure 4.

**Figure 5:**
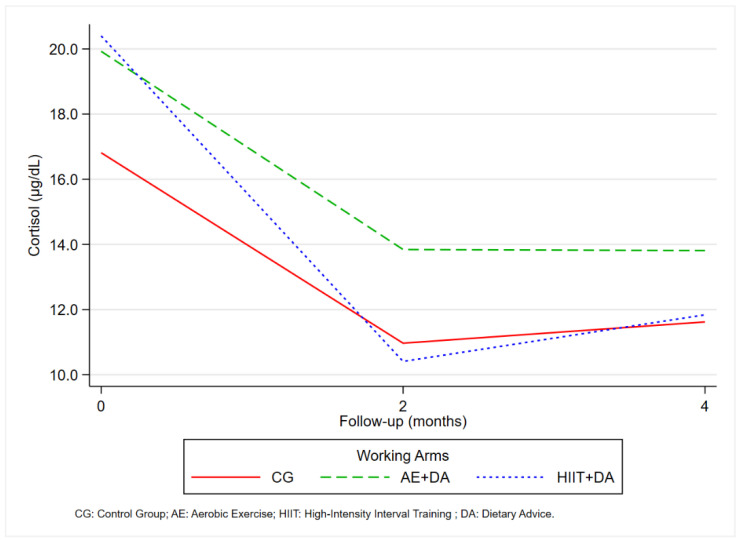
Generalized estimating equation (GEE): Predictive margins of cortisol levels by working arms and time.

The results shown in Figure 5 demonstrate a significant decrease in cortisol levels during the first two months of follow-up, followed by a slight increase from the second month until the end of the study. This trend was observed in all three treatment groups. It is important to note that although cortisol levels had increased by the end of the study, they did not reach the initial levels observed at the beginning of the study.

## 4. Discussion

Lifestyle changes are crucial in the management of MASLD. A Mediterranean diet and regular exercise improve the quality of life and reduce hepatic steatosis [2,11]. In the presence of hepatic steatosis, abnormal levels of circulating cortisol [5] and an often impaired emotional state [10] can often be seen. High cortisol levels promote hepatic lipogenesis and induce insulin resistance, leading to worsening MASLD. In this context, a balanced diet and consistent physical activity do not only improve physical health but can also promote emotional well-being.

In our study, the groups engaged in programmed physical exercise showed a significant reduction in mean circulating cortisol levels, particularly in the HIIT + DA group, where this effect was observed as early as after the first two months. In the AE + DA group, the reduction in cortisol levels was gradual, lasting until the end of the project. Although the HIIT + DA group experienced a slight increase in cortisol levels following the first FU, the overall results remained the best. The statistical model confirmed a significant reduction in cortisol levels, especially in the HIIT group, in the first two months.

After the 4-month intervention, the CAP scores measured by Fibroscan^®^ had decreased significantly from baseline, with severe hepatic steatosis decreasing from 31 to 17 and non-steatosis increasing from 0 to 10. The CAP improvement was observed in all groups but was statistically significant only in the AE + DA group, supported by improvements in body composition and biochemical measures.

Regarding the participants′ emotional state, again, there was an overall improvement, although the results did not reach statistical significance.

Circulating cortisol levels are regulated by the hypothalamic–pituitary–adrenal (HPA) axis and a neuroendocrine feedback loop that can be activated by physiological stimuli, such as stress, high-fat diets, emotional state, and physical activity [48].

Some studies highlight that the MD can actually help reduce cortisol levels by associating this reduction with a decrease in biomarkers of metabolic health (including HbA1c and ALT) [49], as also found in our study.

Although diet can influence cortisol levels, the association between MD and cortisol levels has not yet been extensively explored. There are currently conflicting results in the literature; ours show that simply following MD-based dietary advice can indeed reduce cortisol levels in as soon as 2 months, and the results are even better when combined with regular exercise.

Given the increased energy demand, physical activity is considered a stressor, as it creates an imbalance in homeostasis [48]. The cortisol response varies according to the type and intensity of exercise, as well as the subject′s level of training. Different types of exercise induce different hormonal responses, with an increase in cortisol secretion proportional to the intensity of the stimulus exerted. The results of our study show a greater reduction in circulating cortisol levels in the group performing HIIT, confirming what was already observed by Velasco et al. in overweight/obese subjects [50]. However, our data are discordant with other studies in the literature, highlighting the need to further investigate the relationship between cortisol levels and exercise intensity. Indeed, it is known that after high-intensity exercise, cortisol levels tend to rise in healthy, normal-weight subjects [50]. Different body compositions might influence the hormonal response to intense exercise, and our results suggest that overweight or obese individuals might benefit more from HIIT. An additional hypothesis is that obese individuals exhibit an alteration in the HPA axis [50].

In our study, the DA + HIIT group showed a notable reduction in cortisol levels during the first two months, followed by a slight increase at the end. This initial drop is likely due to the body′s adaptation to physical stress, with reduced hypothalamic–pituitary–adrenal (HPA) axis activity and improved vagal tone from regular exercise. However, the later rise in cortisol could result from reduced exercise intensity, overtraining, or external factors like stress, poor sleep, or seasonal changes. Additionally, participants′ low baseline fitness may have contributed to greater fluctuations in cortisol levels due to heightened sensitivity to exercise adaptations [51].

MD is characterized by the predominant use of extra virgin olive oil as the main source of added fats and regular intake of plant foods, including whole grains, vegetables, legumes, fresh fruits, nuts, seeds, herbs, and spices. These foods improve insulin sensitivity, while components such as monounsaturated fats and polyphenols have anti-inflammatory and antioxidant actions, thus contributing to the reduction in hepatic steatosis and inflammation, as also observed in the results of our study [15].

It is well known that physical activity can help improve many chronic conditions [4]; specifically, it is the first line of intervention for NAFLD management together with diet [11,12,13].

Also, when carried out alone, it can yield important benefits in individuals with MASLD, with positive effects on the control of glucose and lipid metabolism [52]; in fact, it can improve insulin sensitivity in peripheral tissues and the liver and glucose metabolism (or glycemic control in clinically manifest diabetes), slowing the progression of NAFLD. It also reduces systemic inflammation, lowers blood pressure, and improves dyslipidemia [53].

Our results confirm what is already reported in the literature; in fact, in the arms including physical exercise, a reduction in CAP values and an improvement in the hepatic clinical picture were noted, although this was greater in subjects who practiced moderate aerobic physical activity [13]. A previous study had already shown the efficiency of aerobic activity in CAP reduction, even more so when associated with MD, compared with resistance exercise [13]. In our study, moderate-intensity aerobic activity was found to be more effective even than high-intensity interval exercise. There are three main mechanisms involved in the improvement of hepatic steatosis during aerobic activity: activation of lipolysis in different tissues, upregulation of uncoupling protein-1 and peroxisome proliferator-activated receptor γ pathways, and alteration of adipokine levels [25].

After our 4-month study, the group engaged in moderate aerobic exercise also demonstrated a statistically significant reduction in waist circumference, reflecting a decrease in abdominal fat. This effect can be attributed to improved energy balance and increased fat oxidation during and after exercise, particularly in visceral adipose tissue. The reduction in waist circumference is clinically relevant, as it is a key marker of cardiometabolic risk, strongly associated with insulin resistance, inflammation, and other metabolic abnormalities [54].

A reduction in the HOMA-IR index was observed among the same participants, although it did not reach statistical significance. This finding suggests a potential relationship between improvements in anthropometric measures and certain metabolic parameters. While the lack of statistically significant changes in HOMA-IR and other metabolic parameters may seem atypical for an effective 4-month intervention, it is important to note that metabolic adaptations often require longer periods to manifest or may be influenced by interindividual variability.

It is known that nutritional factors and physical activity are closely associated with both improved overall health and perceived well-being [55]. High adherence to MD is correlated with increased feelings of happiness, as it promotes the adoption of a healthy lifestyle and improved perception of quality of life [56]. Physical activity also contributes to the development of self-esteem related to the body, including aspects such as sports competence, physical appearance, stamina, and muscle strength, all of which are positively associated with greater emotional well-being [57]. Although no statistically significant changes emerged, improvements in emotional well-being were observed in all groups compared to baseline levels, with a more pronounced impact in the group that followed a combined HIIT and diet program. This improvement could be attributed to lower starting levels in the HIIT group, making the change more noticeable. In addition, it can be hypothesized that the exercise performed in the group may have motivated the participants by providing psychosocial benefits from group membership [58,59]. Furthermore, all sessions were accompanied by background music, which has been shown to have numerous psychological benefits, including improvements in mood, perception of fatigue, and motivation [60]. These factors combined may have contributed positively to the participants′ emotional state.

It is important to note the high adherence to the project in the groups where physical activity was included. Adherence calculated on the basis of daily attendance taken by the kinesiologists showed high participation. The constant supervision by the kinesiologists certainly helped to keep motivational levels high by ensuring that they provided constant support and supervision both during the walks and in the gym.

However, the study has some limitations, including the difficulty in monitoring dietary adherence and the fact that the happiness questionnaires were administered only at the last FU but not the first FU. This could have given more information in assessing the impact on mood and the possible relationship with the reduction in circulating cortisol levels. In addition, assessments of cortisol levels were made via blood sampling rather than salivary detection, as found in the literature [61]. Despite this, blood sampling was always performed at the same time slot in all FUs.

It would be important in the future to conduct studies that include a personalized diet and provide a food diary to monitor adherence to the nutrition plan.

## 5. Conclusions

In conclusion, our study underscores the importance of MD and physical activity in managing MASLD and reducing circulating cortisol values. Supplementing MD with moderate aerobic exercise led to a significant reduction in hepatic steatosis and circulating cortisol levels; the effects on the latter were particularly evident in the group undergoing the HIIT program. Although improvements in emotional well-being did not reach statistical significance, lifestyle changes still showed positive effects in these subjects. These results suggest that the choice of type of exercise combined with a diet based on MD principles can be tailored to specific goals to optimize the treatment of MASLD. However, further studies are needed to better clarify the relationship between exercise intensity and cortisol levels, considering their potential role in metabolic health.

## Figures and Tables

**Table 1:** FITT parameters of the two different exercise programs.

	Aerobic Exercise	HIIT
Intensity	60–75% HR max	>85% HR max
Type	Aerobic	Aerobic
Time	60 min	50 min
Frequency	4 sessions/week	3 sessions/week

**Table 2:** Example of an aerobic exercise session.

	Time	Exercise
Warm-up	5 min	Joint mobility
Aerobic exercise (walking)	50 min	10 min normal walking30 min sustained walking10 min fast walking
Cool-down	5 min	Stretching

Appendix B shows the progression of the aerobic exercise program.

**Table 3:** Example of an HIIT session.

	Time	Exercise
Warm-up	10 min	Joint mobility + TABATA
HIIT	30 min	TABATA or AMRAP
Cool-down	5/10 min	Stretching

Appendix B shows the progression of the aerobic exercise program.

**Table 4:** Descriptive characteristics by follow-up time.

	Follow-Up Time			
Parameter	Baseline	2 Months	4 Months	*p*-Value ^¥^
N	42	41	39	
Gender **				
Female	23 (55)	22 (54)	21 (55)	0.99
Male	19 (45)	19 (46)	17 (45)	
Age (years) *	51.20 (10.52)	51.67 (10.68)	51.81 (10.39)	0.98
Cortisol (µg/dL) *	16.68 (7.30)	11.63 (4.42)	11.25 (3.53)	<0.001
E (kPa) **	5.5 (4.2–6.4)		5.10 (4.2–5.8)	0.25
CAP (dB/m) ***	299 (279–336)		271 (246–304)	0.001
Fibroscan^®^ cutoff **				
S0 (<248)	0 (0)		10 (26)	0.003
S1 (≥248 <268)	6 (14)		7 (18)	
S2 (≥268 <280)	5 (12)		5 (13)	
S3 (≥280)	31 (74)		17 (44)	
Weight (kg) *	95.60 (15.30)	93.53 (15.11)	92.45 (15.64)	0.64
Waist (cm) *	106.96 (10.02)	104.27 (9.51)	99.42 (13.99)	0.012
BMI (kg/m^2^) *	34.58 (4.06)	33.79 (4.00)	33.27 (4.12)	0.35
BMI categories **				
Overweight	1 (2)	4 (10)	8 (21)	0.13
Obesity	37 (88)	33 (80)	27 (69)	
Severe obesity	4 (10)	4 (10)	4 (10)	
Hips (cm) *	116.20 (8.62)	114.09 (8.83)	111.78 (9.46)	0.090
Glucose (mg/dL) *	99.50 (11.87)	102.15 (14.92)	100.05 (11.25)	0.62
HbA1c (mmol/mol) *	5.57 (0.41)	5.52 (0.44)	5.48 (0.44)	0.67
TC (mg/dL) *	204.89 (41.59)	198.63 (34.94)	194.79 (38.71)	0.50
HDL (mg/dL) *	52.03 (12.78)	45.25 (10.50)	50.05 (11.79)	0.029
LDL (mg/dL) *	134.11 (33.19)	125.59 (28.51)	128.36 (34.03)	0.47
AST (U/L) *	20.99 (8.49)	19.44 (5.81)	18.65 (5.00)	0.29
ALT (U/L) *	25.86 (14.12)	24.29 (11.31)	21.94 (8.01)	0.32
GGT (U/L) *	31.00 (20.05)	25.83 (14.92)	25.07 (17.80)	0.26
TG (mg/dL) *	134.76 (73.38)	128.24 (95.97)	114.36 (44.93)	0.48
ACTH (pg/mL) *	23.10 (14.91)	22.69 (13.29)	18.39 (10.36)	0.22
HOMA-IR *	4.32 (2.02)	4.49 (2.92)	4.17 (2.25)	0.84

^¥^ Continuous variables for time were compared using the Wilcoxon rank sum (2 groups) or Kruskal–Wallis test (>2 groups), while for categorical variables, the Chi2 test was used; * Mean (SD); ** Nr (%); *** Median (IQR). E: Elasticity (Kilopascal); CAP: Controlled Attenuation Parameter FibroScan^®^; E (kPa): Elasticity (Kilopascal); BMI: Body Mass Index; HbA1c: Glycated Hemoglobin; TC: Total Cholesterol; HDL: High-Density Lipoprotein; LDL: Low-Density Lipoprotein; AST: Aspartate Transaminase; ALT: Alanine Amino Transferase; GGT: Gamma Glutamyl Transferase; TG: Triglycerides; ACTH: Adrenocorticotropic Hormone; HOMA-IR: Homeostasis Model Assessment for Insulin Resistance.

**Table 5:** Descriptive characteristics by follow-up time and working arms.

				Working Arms				
		CG (Diet)		AE + DA		HIIT + DA		All Arms
Parameter	Time	Central Values	*p*-Value ^†^	Central Values	*p*-Value ^†^	Central Values	*p*-Value ^†^	*p*-Value ^¥^
N		17		12		13		
Cortisol (µg/dL) *								
	Baseline	15.82 (6.83)		17.20 (6.22)		17.34 (9.09)		0.82
	2 months	12.56 (5.40)	0.060	12.30 (3.17)	0.021	9.83 (3.55)	0.006	0.21
	4 months	11.25 (3.52)		12.16 (3.22)		10.63 (3.87)		0.62
CAP (dB/m) **								
	Baseline	297 (278–335)		326 (296–335)		293 (268–341)		0.39
	4 months	278 (245–302)	0.062	265 (246–295)	0.005	275 (254–329)	0.32	0.70
E (kPa) **								
	Baseline	5.8 (5.2–6.9)		5.85 (5.05–6.6)		5.40 (4.00–5.70)		0.26
	4 months	5.5 (4.3–6.7)	0.45	5.4 (4.1–5.9)	0.25	4.60 (4.20–5.00)	0.28	0.21
Weight (kg) *								
	Baseline	96.52 (15.74)		97.18 (20.29)		92.92 (8.98)		0.75
	2 months	95.11 (15.82)	0.96	94.89 (20.07)	0.60	90.33 (8.70)	0.80	0.66
	4 months	96.22 (16.63)		89.08 (17.96)		90.96 (12.33)		0.48
Waist (cm) *								
	Baseline	107.32 (8.43)		108.12 (12.85)		105.42 (9.65)		0.79
	2 months	105.12 (9.14)	0.67	105.73 (11.29)	0.045	101.92 (8.67)	0.20	0.56
	4 months	104.73 (9.29)		92.40 (21.54)		99.23 (7.21)		0.082
BMI (kg/m^2^) *								
	Baseline	34.28 (4.08)		33.53 (3.03)		35.95 (4.75)		0.31
	2 months	33.79 (4.28)	0.95	32.47 (3.01)	0.21	34.91 (4.30)	0.58	0.34
	4 months	33.96 (4.68)		31.29 (2.81)		34.15 (4.09)		0.17
Hips (cm) *								
	Baseline	116.53 (9.43)		114.33 (8.67)		117.50 (7.81)		0.65
	2 months	114.58 (9.84)	0.58	111.55 (8.76)	0.36	115.62 (7.61)	0.36	0.52
	4 months	112.80 (10.77)		109.05 (8.61)		112.92 (8.75)		0.54
Glucose (mg/dL) *								
	Baseline	100.65 (11.89)		100.75 (15.34)		96.85 (8.14)		0.64
	2 months	106.88 (19.19)	0.43	103.36 (12.18)	0.60	94.92 (6.14)	0.83	0.087
	4 months	105.36 (9.63)		96.97 (13.80)		96.05 (9.22)		0.055
HbA1c (mmol/mol) *								
	Baseline	5.59 (0.43)		5.53 (0.52)		5.57 (0.28)		0.94
	2 months	5.55 (0.50)	0.95	5.45 (0.52)	0.94	5.53 (0.29)	0.44	0.86
	4 months	5.54 (0.41)		5.47 (0.64)		5.42 (0.32)		0.79
TC (mg/dL) *								
	Baseline	201.08 (45.19)		213.08 (44.33)		202.31 (35.93)		0.73
	2 months	195.53 (36.70)	0.92	207.91 (44.73)	0.53	194.85 (22.31)	0.74	0.60
	4 months	198.12 (39.46)		190.30 (52.19)		194.05 (28.86)		0.89
HDL (mg/dL) *								
	Baseline	53.69 (15.49)		52.48 (11.20)		49.45 (10.57)		0.67
	2 months	47.30 (10.91)	0.39	45.85 (10.15)	0.21	42.08 (10.27)	0.15	0.40
	4 months	49.55 (14.06)		54.03 (11.64)		47.85 (8.87)		0.48
LDL (mg/dL) *								
	Baseline	132.62 (32.92)		141.45 (37.93)		129.28 (30.29)		0.65
	2 months	125.73 (28.67)	0.73	131.32 (37.65)	0.52	120.57 (19.38)	0.66	0.67
	4 months	133.60 (30.99)		120.58 (48.85)		127.71 (26.15)		0.67
AST (U/L) *								
	Baseline	19.26 (6.89)		23.75 (10.22)		20.69 (8.68)		0.38
	2 months	17.65 (4.53)	0.68	23.45 (7.81)	0.24	18.38 (3.69)	0.67	0.022
	4 months	18.51 (4.17)		18.07 (4.17)		19.22 (6.53)		0.87
ALT (U/L) *								
	Baseline	24.65 (10.23)		32.92 (21.69)		20.92 (5.65)		0.092
	2 months	22.71 (10.11)	0.78	29.64 (16.00)	0.32	21.85 (6.34)	0.95	0.18
	4 months	22.69 (6.16)		21.48 (9.25)		21.38 (9.49)		0.90
GGT (U/L) *								
	Baseline	34.82 (23.47)		30.67 (21.00)		26.31 (13.81)		0.52
	2 months	29.18 (15.57)	0.38	21.64 (8.96)	0.20	25.00 (17.88)	0.97	0.42
	4 months	26.27 (10.59)		20.29 (6.85)		27.00 (27.66)		0.66
TG (mg/dL) *								
	Baseline	102.17 (40.31)		150.00 (71.63)		163.31 (94.39)		0.050
	2 months	98.41 (36.46)	0.91	128.91 (60.81)	0.32	166.69 (151.60)	0.65	0.16
	4 months	103.74 (32.95)		108.16 (48.86)		130.92 (52.26)		0.26
ACTH (pg/mL) *								
	Baseline	22.51 (10.08)		28.61 (23.82)		18.72 (6.49)		0.25
	2 months	23.22 (13.19)	0.72	23.21 (18.84)	0.47	21.55 (7.70)	0.26	0.94
	4 months	20.00 (11.68)		18.05 (11.89)		16.77 (7.87)		0.72
HOMA-IR *								
	Baseline	4.11 (1.88)		4.84 (2.71)		4.12 (1.44)		0.59
	2 months	5.10 (3.77)	0.61	3.89 (2.41)	0.42	4.21 (1.92)	0.98	0.53
	4 months	4.66 (2.70)		3.51 (1.83)		4.07 (1.94)		0.48

* Mean (SD); ** Median (IQR). *p*-value: ^¥^ Variables by working arms are compared using the Wilcoxon rank sum (2 groups) or Kruskal–Wallis test (>2 groups); ^†^ Variables by working arms and time are compared using the Wilcoxon rank sum (2 times) or Kruskal–Wallis test (3 times). DA: Dietary Advice; CG: Control Group; AE: Aerobic Exercise; HIIT: High-Intensity Training. CAP: Controlled Attenuation Parameter FibroScan^®^; BMI: Body Mass Index; HbA1c: Glycated Hemoglobin; TC: Total Cholesterol; HDL: High-Density Lipoprotein; LDL: Low-Density Lipoprotein; AST: Aspartate Transaminase; ALT: Alanine Amino Transferase; GGT: Gamma Glutamyl Transferase; TG: Triglycerides; ACTH: Adrenocorticotropic Hormone; HOMA-IR: Homeostasis Model Assessment for Insulin Resistance.

**Table 6:** Generalized estimating equation (GEE): Expected values for cortisol levels by time and working arm.

	β	SE(β)	95% CI
Follow-Up:			
Baseline	0.0		
2 months	−5.85 **	1.54	−8.88; −2.82
4 months	−4.84 **	1.58	−7.94; −1.75
Working Arm:			
CG	0.00		
AE + DA	3.73	2.96	−2.08; 9.54
HIIT + DA	4.13	2.76	−1.27; 9.53

** *p* < 0.001. Model adjusted for gender, age, ALT, AST, ACTH, and waist circumference. CG: Control Group; AE: Aerobic Exercise; HIIT: High-Intensity Training; DA: Dietary Advice.

**Table 7:** Generalized estimating equation (GEE): Contrasts between working arms and time from baseline.

Contrast	β	SE(β)	95% CI
Time@Working Arms			
(2 ms vs. t0) CG	−5.85 **	1.55	−8.88; −2.82
(4 ms vs. t0) CG	−4.84 **	1.58	−7.94; −1.75
(2 ms vs. t0) DA + AE	−5.86 *	2.40	−10.56; −1.16
(4 ms vs. t0) DA + AE	−5.18 *	2.55	−10.17; −0.19
(2 ms vs. t0) DA + HIIT	−11.10 **	1.95	−14.9; −7.28
(4 ms vs. t0) DA + HIIT	−8.83 **	2.07	−12.89; −4.78

** *p* < 0.001, * *p* < 0.05. CG: Control Group; AE: Aerobic Exercise; HIIT: High-Intensity Training; DA: Dietary Advice; ms: Months. Model adjusted for gender, age, ALT, AST, ACTH, and waist circumference.

## Data Availability

Data are available upon reasonable request.

## Supplementary Materials

The following supporting information can be downloaded at: https://www.mdpi.com/article/10.3390/nu16234225/s1, Table S1: Distribution of participants according to follow-up time and working arms for the eight subscales of the SF36 questionnaire for the five variables related to the “emotional well-being” group and the Subjective Happiness Scale (SHS).

## Appendix A

Dietary guidelines for healthy eating (CREA).

## Appendix B

### Appendix B.1. Exercise Progression

#### Appendix B.1.1. Aerobic Exercise Progression

Before starting the first training session, participants were informed about the heart rate range to maintain during all walks, personalized with the Tanaka formula (ref this formula). Researchers explained that in addition to heart rate monitoring, the Talk Test and the Borg scale will be used to measure the pace and perception of fatigue. In the first outing, an initial part was dedicated to explaining the correct walking technique and then continued with the established program.

The first two weeks were dedicated to a conditioning phase, designed to progressively increase the subjects′ tolerance. The session was divided into three blocks of 15 min of walking punctuated by 2 min of recovery to allow the group to regroup and ask about the level of perceived fatigue.

In the following month and a half, walking was divided into two blocks of 25 min, with a single recovery of 2 min.

In the last month and a half, walking was performed for 50 consecutive minutes, varying the walking pace and the route based on the differences in altitude. Recovery was performed only if necessary.

	**Aerobic Session**	**Time**	**Frequency**
Conditioning	Warm-up + joint mobility 3 sets 15′ walks Intensity: HR 60–75%	5′ 45′ 2′ rest between the sets	4 times per week
Mesocycle 1	Warm-up + joint mobility 2 sets 15′ walks Intensity: HR 60–75%	5′ 45′ 2′ rest between the sets	4 times per week
Mesocycle 2	Warm-up + joint mobility Continuous walk Intensity: HR 60–75%	5′ 50′	4 times per week

#### Appendix B.1.2. HIIT Progression

The first two weeks of work were dedicated to a conditioning phase (six workouts). In this first period, we worked with natural load, using the TABATA protocol adapted to the participants, alternating work times of 20 s with recovery times of 20 s.

In the first mesocycle (14 workouts), we intensified the work by reducing the rest time (20” work and 10” rest) both in the warm-up phase and in the central phase. From this mesocycle onwards, we began to work not only with natural load but also with the use of weights.

In the second mesocycle (15 workouts), we increased the work time, reaching 30” of work and 10” of rest.

In the third mesocycle (15 workouts), we concluded the progression of work times, reaching 40” of work and 15” of recovery and adding one session per week dedicated to AMRAP (as many reps/rounds as possible).

	**Tabata Block + Intensity**	**Time**	**Total HIIT Time**	**Frequency**
Conditioning	TABATA: 20″ work + 20″ rest Intensity: HR 50–60% 4 sets of TABATA: 20″ work + 20″ rest Intensity: HR > 85%	10′ work 3′ rest 20′ work	30′	3 times per week
Mesocycle 1	TABATA: 20″ work + 10″ rest Intensity: HR 50–60% 5 sets of TABATA: 20″ work + 10″ rest Intensity: HR > 85%	8′ work 3′ rest 20′ work	28′	3 times per week
Mesocycle 2	TABATA: 20″ work + 10″ rest Intensity: HR 50–60% 5 sets of TABATA: 30″ work + 10″ rest Intensity: HR > 85%	8′ work 3′ rest 22′ work	30′	3 times per week
Mesocycle 3	TABATA: 20″ work + 10″ rest Intensity: HR 50–60% 4 sets of TABATA: 40″ work + 15″ rest Intensity: HR > 85% 2 sets of AMRAP	8′ work 3′ rest 28′ work 3‘ rest between 2 TABATA 12′ work 3′ rest between AMRAP 12′ work	36′ 32′	3 times per week 2 times per week * Once per week **
* On Monday and on Friday, ** On Wednesday.				

Examples of some exercises used during the HIIT session.

**Training Session**	**Examples****of Some****Exercises**
Warm-up 1 (joint mobility) Warm-up 2 (TABATA)	Ankle circles, wrist, march in place, arm circles, bird/dog, t-spine rotation, child pose Jumping jacks, run, air squat, light skip, crunch, bridge, plank
TABATA	Weighted squat, thruster, burpees, lunges, lateral raises, step up
AMRAP	12 lunges 15 Russian swings 10 crunches with kettlebell 16 mountain climbers
COOL-DOWN	Standing upper body stretching Sitting or lying lower body stretching
