# Supplementary material for: Healthy Lifestyle Changes Improve Cortisol Levels and Liver Steatosis in MASLD Patients: Results from a Randomized Clinical Trial
Source: Nutrients. 2024 Dec 6;16(23):4225. doi: 10.3390/nu16234225 (PMC11644361; doi:10.3390/nu16234225)
Supplement: Supplementary file 1 [file nutrients-16-04225-s001.zip › nutrients-3343429-supplementary.pdf]

Supplementary Tables

Table S1. Distribution of participants according to follow-up time and working arms for the eight subscales of the SF36 questionnaire, for the five variables related to the 'emotional well-being' group and the Subjective Happiness Scale (SHS)

		Working Arms						
	All sample	CG	AE+DA	HIIT+DA	*p-value*$^{\$}$	*p-value*†	*p-value*$^{\psi}$	*p-value*$^{¥}$
		Median (IQR)	Median (IQR)	Median (IQR)				
SHS								
T base	5.25 (4.50-5.75)	5.38 (4.75-5.75)	5.25 (4.62-6.12)	4.00 (3.75-5.50)	0.21	0.68	0.64	0.37
T 4 months	5.50 (4.75-6.00)	5.50 (4.75-6.00)	5.25 (4.75-6.00)	5.25 (4.50-6.00)	0.92			
Physical functioning								
T base	95.00 (75.00-95.00)	95.00 (85.00-95.00)	92.50 (72.50-97.50)	90.00 (80.00-95.00)	0.89	0.71	0.24	0.14
T 4 months	95.00 (90.00-100.00)	90.00 (80.00-95.00)	95.00 (90.00-100.00)	95.00 (90.00-100.00)	0.26			
Role limitations due to physical health								
T base	100.00 (75.00-100.00)	100.00 (75.00-100.00)	100.00 (100.00-100.00)	100.00 (75.00-100.00)	0.30	0.91	0.64	0.64
T 4 months	100.00 (100.00-100.00)	100.00 (75.00-100.00)	100.00 (100.00-100.00)	100.00 (75.00-100.00)	0.41			
Role limitations due to emotional problems								
T base	100.00 (66.67-100.00)	100.00 (66.67-100.00)	100.00 (100.00-100.00)	100.00 (66.67-100.00)	0.45	0.98	0.53	0.13
T 4 months	100.00 (100.00-100.00)	100.00 (66.67-100.00)	100.00 (100.00-100.00)	100.00 (100.00-100.00)	0.30			
Energy/fatigue								
T base	60.00 (50.00-75.00)	55.00 (50.00-70.00)	70.00 (55.00-75.00)	60.00 (50.00-65.00)	0.22	0.58	0.73	0.25
T 4 months	65.00 (55.00-75.00)	60.00 (50.00-75.00)	70.00 (60.00-80.00)	65.00 (55.00-80.00)	0.41			
Emotional well-being								
T base	74.00 (60.00-80.00)	68.00 (60.00-80.00)	76.00 (64.00-82.00)	72.00 (52.00-76.00)	0.58	0.83	0.78	0.76
T 4 months	72.00 (64.00-80.00)	72.00 (64.00-84.00)	76.00 (64.00-84.00)	72.00 (56.00-76.00)	0.50			
Social functioning								
T base	87.50 (62.50-87.50)	87.50 (75.00-87.50)	75.00 (56.25-87.50)	87.50 (62.50-87.50)	0.42	0.84	0.61	0.57
T 4 months	75.00 (62.50-87.50)	87.50 (75.00-100.00)	75.00 (62.50-100.00)	87.50 (62.50-87.50)	0.50			
Pain								

T base	83.75 (67.50-100.00)	90.00 (67.50-100.00)	90.00 (72.50-95.00)	67.50 (67.50-90.00)	0.44	0.65	0.29	0.81
T 4 months	90.00 (67.50-100.00)	90.00 (67.50-100.00)	90.00 (87.50-100.00)	67.50 (55.00-90.00)	0.045			
General Health								
T base	65.00 (55.00-75.00)	65.00 (55.00-75.00)	62.50 (47.50-65.00)	75.00 (55.00-75.00)	0.29	0.13	0.19	0.70
T 4 months	70.00 (60.00-80.00)	75.00 (60.00-80.00)	65.00 (60.00-85.00)	75.00 (65.00-75.00)	0.84			
In general. would you say your health is								
T base	50.00 (50.00-50.00)	50.00 (50.00-50.00)	50.00 (25.00-50.00)	50.00 (50.00-75.00)	0.40	0.34	0.030	0.24
T 4 months	50.00 (50.00-75.00)	50.00 (50.00-75.00)	75.00 (50.00-75.00)	75.00 (50.00-75.00)	0.85			
Have you been a very nervous person								
T base	80.00 (60.00-80.00)	80.00 (60.00-80.00)	80.00 (60.00-80.00)	80.00 (60.00-80.00)	0.90	0.74	0.92	0.61
T 4 months	80.00 (60.00-80.00)	80.00 (60.00-80.00)	80.00 (60.00-80.00)	80.00 (60.00-80.00)	0.95			
Have you felt so down in the dumps that nothing could cheer you up								
T base	100.00 (80.00-100.00)	100.00 (80.00-100.00)	100.00 (80.00-100.00)	80.00 (80.00-100.00)	0.66	0.63	0.76	0.87
T 4 months	100.00 (80.00-100.00)	100.00 (80.00-100.00)	100.00 (80.00-100.00)	80.00 (80.00-100.00)	0.42			
Have you felt calm and peaceful								
T base	60.00 (40.00-80.00)	60.00 (60.00-80.00)	60.00 (40.00-80.00)	40.00 (40.00-60.00)	0.30	0.43	0.46	0.77
T 4 months	60.00 (40.00-80.00)	60.00 (40.00-80.00)	80.00 (40.00-80.00)	60.00 (20.00-60.00)	0.35			
Do you have a lot of energy								
T base	60.00 (40.00-60.00)	60.00 (40.00-60.00)	60.00 (40.00-80.00)	40.00 (20.00-60.00)	0.29	0.74	0.24	0.061
T 4 months	60.00 (40.00-80.00)	40.00 (40.00-60.00)	80.00 (40.00-80.00)	60.00 (60.00-80.00)	0.20			
Have you felt downhearted and blue								
T base	80.00 (60.00-80.00)	80.00 (60.00-100.00)	80.00 (60.00-90.00)	80.00 (60.00-80.00)	0.80	0.87	0.27	0.23
T 4 months	80.00 (60.00-80.00)	80.00 (60.00-100.00)	80.00 (80.00-100.00)	80.00 (60.00-80.00)	0.057			
Did you feel worn out								
T base	80.00 (60.00-100.00)	80.00 (60.00-100.00)	80.00 (70.00-100.00)	80.00 (60.00-80.00)	0.78	0.75	1.00	0.45
T 4 months	80.00 (60.00-80.00)	80.00 (60.00-100.00)	80.00 (80.00-80.00)	80.00 (60.00-80.00)	0.11			
Have you been a happy person								
T base	60.00 (40.00-80.00)	60.00 (40.00-80.00)	60.00 (50.00-80.00)	60.00 (40.00-80.00)	0.67	1.00	0.95	0.63
T 4 months	60.00 (40.00-80.00)	60.00 (40.00-80.00)	60.00 (40.00-80.00)	60.00 (40.00-80.00)	0.85			

p-value: [$] Working Arms and time of Follow-up are compared using the Kruskal-Wallis; [†]Wilcoxon rank-sum between the parameters recorded at the two times for the diet; [ψ]Wilcoxon rank-sum between the parameters recorded at the two times for the AE+DAt; [¥]Wilcoxon rank-sum between the parameters recorded at the two times for the HIIT+DA. DA: Dietary Advice; CG: Control Group; AE: Aerobic Exercise; HIIT: High-Intensity Training.
